# Determinants of sustainable quality improvement for operating room clean air systems: a two-phase sequential qualitative study in Dutch hospitals

**DOI:** 10.1186/s12913-026-14358-9

**Published:** 2026-03-30

**Authors:** Egid M. van Bree, Erin N. A. Cornelisse, Roberto A. A. L. Traversari, Evelyn A. Brakema, Nicole D. Bouvy, Rianne M. J. J. van der Kleij

**Affiliations:** 1 Maastricht University, Maastricht, the Netherlands; 2 Department of Surgery, Leiden University Medical Centre, Albinusdreef 2, Leiden, 2333 ZA the Netherlands; 3 Centre for Sustainable Healthcare, Amsterdam UMC, Amsterdam, the Netherlands; 4 Organisation for Applied Scientific Research, TNO, Delft, the Netherlands; 5 Department of Public Health and Primary Care/Campus the Hague, Leiden University Medical Centre, Leiden, the Netherlands

**Keywords:** Sustainable healthcare, Environmental sustainability, Healthcare quality improvement, Implementation science, Heating, ventilation, and air conditioning

## Abstract

**Background:**

To address healthcare’s polluting environmental impacts, environmental sustainability is increasingly recognised as a key domain of healthcare quality improvement. Heating, ventilation, and air conditioning (HVAC) systems are important targets for environmental sustainability-oriented quality improvement, causing 70–90% of operating room (OR) energy consumption and incurring substantial amounts of greenhouse gas emissions. Safe and effective energy-saving measures exist yet are infrequently implemented in practice. This study aims to identify key implementation determinants for HVAC-related energy saving in the OR and to explore their prioritisation and interrelation.

**Methods:**

We performed a two-phase sequential qualitative study in Dutch academic and general hospitals. Clinicians, engineers, managers, and infection prevention specialists with HVAC- and OR-related expertise were recruited using purposive sampling. First, we conducted semi-structured interviews based on the Consolidated Framework for Implementation Research. Findings were compiled into a shortlist of potential barriers and facilitators of implementation. Next, we performed combined ranking exercises and focus groups in different hospitals. Recordings were transcribed verbatim, coded by two researchers using a deductive-inductive approach, and analysed using Thematic Analysis. Ranking outcomes and focus group findings were combined to map key implementation determinants and processes.

**Results:**

We included 42 participants, based on expert interviews (*n* = 12) and focus groups (*n* = 32, two overlapped) in five hospitals. Prioritised barriers were technical and organisational, linked to hospitals’ size and structure, adjustability of the current HVAC system, and availability of in-house expertise. Limited HVAC-related knowledge and equivocal beliefs regarding HVAC’s contribution to infection prevention shaped perceived feasibility of energy-saving measures and compatibility with OR workflows. Prioritised facilitators were the evidence base for energy-saving measures, organisations’ relative priority to save energy, and presence of a dedicated implementation lead. Both bottom-up and top-down approaches had initiated HVAC adjustment processes in studied hospitals.

**Conclusions:**

Implementation of HVAC-related energy-saving measures in the OR is hampered by perceived implementation complexity, technical challenges, and individual knowledge gaps and beliefs. Dedicated multidisciplinary workgroups, managerial priority for energy saving, and collaboration of motivated individuals with complementary expertise and influence appear to support successful local implementation.

**Supplementary Information:**

The online version contains supplementary material available at 10.1186/s12913-026-14358-9.

## Background

In the face of environmental degradation with profound consequences for human and planetary health [1, 2], there is growing momentum in the healthcare sector to reduce its polluting environmental impact [3]. Accounting for 4–5% of global carbon emissions and up to 13% of natural resource use in the Netherlands [4, 5], environmental sustainability requires to be addressed as an integral element of future-proof healthcare. Contemporary healthcare quality frameworks therefore underline the importance to consider environmental outcomes alongside criteria such as effectiveness and safety [6–8]. Consequently, environmental sustainability is increasingly recognised as a key domain of healthcare quality improvement (QI) [9, 10]. Especially in the operating room (OR), a major contributor to hospitals’ resource use and greenhouse gas emissions [11, 12], QI initiatives to reduce environmental impact are crucial.

Heating, ventilation, and air-conditioning (HVAC) systems are important targets for environmental sustainability in the OR. Causing 70–90% of the OR’s energy consumption [11, 13], their operation incurs substantial amounts of greenhouse gas emissions through incineration of fossil fuels. HVAC systems circulate up to 12,500 m^3^h^−1^ of air which partially needs to be heated, cooled, and (de)humidified to predefined setpoints. Multiple HVAC-related energy-saving measures are reported as effective strategies to reduce its environmental harm and costs, without affecting safety [14–17]. These include setbacks outside working hours, widening relative humidity setpoints, and reducing volumes of (outdoor) air circulation – yielding estimated energy savings up to 93%. [15] Yet, implementation of these strategies varies greatly across Dutch hospitals, with insufficient comprehension of underlying causes.

To the best of our understanding, implementation determinants of HVAC-related energy saving to improve OR’s environmental sustainability have not been studied. Operation of HVAC systems has primarily been viewed through a lens of safety. National guidelines frequently advise high-volume air circulation to achieve ‘ultra clean’ air conditions [18–20], mostly based on evidence regarding arthroplasty of major joints [21, 22]. However, evidence for high-volume HVAC to reduce the risk of surgical site infections for other types of surgeries is equivocal. Systematic reviews nor recent observational studies demonstrated a significant benefit of ultra clean HVAC systems [23–25], resulting in a conundrum of inconclusive evidence for absent to marginal safety returns [20, 22, 26, 27]. Reassessment through environmental sustainability-focussed QI is therefore highly needed.

Given the translational gap between daily practice and the clear environmental and financial benefit, the aim of this study is to identify key determinants influencing the local implementation process of HVAC-related energy saving in Dutch ORs. Particularly, we aimed to explore prioritisation and interrelation of determinants to allow for targeted, context-informed QI efforts.

## Methods

### Study design

We conducted a two-phase sequential qualitative study in Dutch hospitals. Phase one consisted of semi-structured expert interviews based on the Consolidated Framework for Implementation Research (CFIR) [28]. We compiled findings into a shortlist of potential implementation barriers and facilitators. In phase two, we performed combined ranking exercises and focus groups in different hospitals. Recordings were transcribed verbatim, coded independently by two researchers using a deductive-inductive approach, and analysed using Thematic Analysis [29]. Hospitals’ HVAC-related energy-saving implementation processes were analysed by (re)matching top-ranked barriers and practiced implementation strategies to the Expert Recommendations for Implementing Change (ERIC) compilation [30]. We followed the COREQ guidelines for reporting of qualitative studies where applicable [31]. Ethical approval was waived by the institutional review board (file number:2024–0478). Participants gave informed consent for study participation.

### Setting

This study was executed in the Netherlands, a country with a decentralised health system of private insurers and private providers. Dutch hospitals are independent organisations, able to equip and organise their ORs according to local preferences – given that they are certified to meet national technical and professional standards. For example, the 2022 guideline of the Dutch Federation of Medical Specialists details several minimum HVAC and air quality requirements for patient safety, differentiating between ‘ultra clean’ and ‘clean’ ORs [18]. Considering the equivocal evidence regarding high-volume HVAC and the multitude of aseptic measures in modern ORs, only arthroplasty of major joints have an ultra clean air recommendation in the Dutch guideline.

Notwithstanding the possibility for OR HVAC differentiation, most hospitals perform all surgeries in ultra clean air conditions – whereas arthroplasty of major joints only make up 5–10% of their case load [15]. Previous HVAC guidelines’ tendency to maximise risk reduction have led to the adoption of high-volume unidirectional flow HVAC systems in most Dutch hospitals. However, their design and way of operating can vary, leading to substantial variation in practice across hospitals. Recent years have seen growing attention for environmentally sustainable healthcare in the Netherlands, supported by the widely adopted ‘Green Deal for Sustainable Healthcare’ and the inception of a ‘National Network for Green ORs’ [32, 33]. Consequently, interest in OR energy saving has increased among clinicians and other hospital staff – although no supporting figures exist.

## Phase one: expert interviews

### Participants

We invited participants with HVAC- and OR-related expertise from hospitals and independent HVAC service organisations across the country. Participants received an invitational email from the investigator and, when willing to participate, were scheduled for an online or in-person interview, chosen at their convenience. Based on suggestions of a national OR HVAC expert group [33], we purposively sampled participants with diverse backgrounds [34], including clinicians, engineers, OR managers, and infection prevention specialists to account for different perspectives. We included at least two participants per discipline with HVAC-related implementation experience in academic or general hospitals, which we considered to suffice for an initial overview. This could include engineers from specialised HVAC service organisations, who generally serve a great number of hospital organisations.

### Data collection and analysis

Expert interviews (30–45 minutes) were semi-structured, starting with an open question followed by a series of questions to probe for implementation determinants of HVAC-related energy saving measures. Probing questions were based on CFIR constructs [28], using the framework’s structure to explicitly inquire which determinants applied within each of the CFIR domains. We instructed participants to base their answers on their own experience and refrain from prioritising. One investigator performed the interviews and took notes of responses to the open and probing questions. The interview guide is available from Supplement A.

All expert interview notes were coded in MAXQDA software (Verbi GmBH, Germany), using a CFIR-derived deductive-inductive approach – grounded in critical realism [35]. In line with the probing questions, codes were assigned per CFIR domain and construct. The codebook and findings were repeatedly discussed with a second investigator and compiled into a shortlist of implementation determinants for HVAC-related energy saving measures. We verified completeness of the final shortlist with the expert interview participants and with other participants directly prior to the ranking exercise.

## Phase two: ranking exercises and focus groups

### Participants

We purposively sampled participants from a diverse group of hospitals [34], containing academic and large to small-sized centres. Again based on suggestions of a national HVAC expert group [33], we contacted hospitals with known difficulty or success to implement HVAC energy saving over the past two years. The investigator approached one contact person per hospital and collaboratively invited local stakeholders involved in HVAC-related decision-making for an on-site session, striving to include at least one engineer, OR manager, infection prevention specialist, and clinician – if involved. Once multiple stakeholders had been identified, the investigator once more inquired whether additional stakeholders should be invited to accurately understand HVAC-related decision-making in the hospital. Participants included in the first research phase were again allowed to participate.

### Data collection

The ranking exercises and focus groups (60–90 minutes combined) started with an individual ranking of the shortlisted implementation determinants, followed by a group discussion of the live-computed mean rankings and determinant interrelations. Two investigators conducted the sessions, introducing the subject and guiding the ranking exercise and focus group discussion. Participants were preferably on-site, with a possibility of a hybrid or online session if necessary. During hybrid sessions, online participants dialled in via a secure video connection on their device of choice (Microsoft Teams, USA), supporting full participation in discussions via videoconferencing equipment in the focus group room. Online participation in ranking exercises was facilitated by providing the shortlist of implementation determinants and participation link via email.

Rankings were performed using online polling software (Mentimeter AB, Sweden), allowing participants to freely divide 100 points over barriers and facilitators separately, resembling their importance. Consecutive focus group discussions were audio-recorded and transcribed verbatim using MAXQDA software. The investigators made individual pre- and post-session research notes, including observations and informal conversations supporting triangulation in data analysis. We continued data collection until data saturation was achieved and no new themes emerged. Summaries of key findings per site were shared with participants for validation. Study materials are available from Supplement A.

### Data analysis

Determinant ranking outcomes were reported descriptively, identifying key barriers and facilitators based on determinants’ assigned total score across sessions, their top five ranking frequency per profession, and their top five ranking frequency per organisation. Analysis and visualisation were performed using R v4.5.1.

Focus group transcripts were independently coded by two investigators, using the same CFIR-derived deductive-inductive approach as the expert interviews, and discussed after every transcript until consensus was reached. We identified main themes and interrelations using Thematic Analysis [29], considering (co-)occurrences of codes in transcripts, participants’ comments regarding their interrelation, and triangulation based on ranking outcomes and research notes. Co-authors were involved in iterative meetings to validate the interpretation and structuring of findings.

Top-ranked barriers and facilitators were re-mapped to underlying CFIR domains and constructs to facilitate matching with relevant ERIC implementation strategies, based on published guidance [36]. We compared endorsed implementation strategies to those practiced and suggested throughout focus groups, connecting key determinants and strategies to main themes and different hospitals’ level of HVAC-related energy saving. Findings were charted into an implementation process map.

### Reflexivity statement

The research team consisted of individuals with diverse backgrounds (medicine, healthcare management, engineering, implementation science) and varying levels of expertise. The investigator who conducted the interviews and focus groups (EMvB) is generally well-versed and well-connected in the Dutch sustainable healthcare movement and knew several of the participants prior to their participation in this study. Several co-authors are part of the same movement (RAALT, EAB, NDB). Whereas this background was particularly helpful in study design, contextualisation, and participant recruitment, it may have unintendedly influenced the data collection and analysis in favour of environmental sustainability. Involvement of a junior researcher unfamiliar with the subject (ENAC) and a supervisor outside of the sustainable healthcare movement (RMJJvdK), helped reduce such bias in data collection, analysis, and reporting.

## Results

Between December 2024 and May 2025, we included 42 participants, based on expert interviews (*n* = 12) and focus groups (*n* = 32) in five hospitals. Most study participants were engineers, and two participants took part in both research phases (Table 1). At least one OR manager, infection prevention specialist, engineer, and clinician with a direct relationship to HVAC operation were included – except for general hospital 3 where no clinicians were involved currently. Participating hospitals varied in their level of HVAC-related energy saving, including no implementation of energy-saving measures and current exploration of ‘ultra clean’ and ‘clean’ OR differentiation. Expert interviews yielded a shortlist of 22 implementation determinants, reported in Supplement B.

**Table 1:** Participant overview of expert interviews and ranking/focus group sessions

	Participant identifier	Participant characteristics (years of working experience)	Workplace
Expert interview 1	P1 P2	Female, anaesthetist (>15) Male, engineer (>15)	Academic hospital Research organisation
Expert interview 2	P3	Female, microbiologist (>15)	Academic hospital
Expert interview 3	P4	Male, engineer (>15)	HVAC service organisation
Expert interview 4	P5	Male, engineer (>10)	HVAC service organisation
Expert interview 5	P6	Female, IP specialist (>15)	General hospital
Expert interview 6	P7 P8	Male, OR manager (>5) Female, IP specialist (>5)	General hospital General hospital
Expert interview 7	P9 P10	Female, resident anaesthetist (<5) Female, anaesthetist (>15)	Academic hospital Academic hospital
Expert interview 8	P11	Female, surgeon (>15)	Academic hospital
Expert interview 9	P12	Female, OR manager (>10)	Academic hospital
Focus group 1 *on-site*	P13 P14 P15 P16	Female, IP specialist (>10) Female, anaesthetist (>15) Male, engineer (>15) Male, OR manager (>15)	General hospital 1 *Energy-saving measures: SB, HR, exploring RC*
Focus group 2 *hybrid*	P17 P18 P19 P20 P21 P22 P23	Male, engineer (>10) Female, OR manager (<5) Female, resident IP specialist (<5) Female, IP specialist (>15) Male, engineer (>15) Male, engineer (>5) Male, engineer (>5)	General hospital 2 *Energy-saving measures: none*
Focus group 3 *on-site*	P24 P3 P25 P26 P27	Male, engineer (>5) Female, microbiologist (>15) Male, engineer (>15) Male, engineer (>15) Male, OR manager (>5)	Academic hospital 1 *Energy-saving measures: SB, HR*
Focus group 4 *on-site*	P28 P9 P29 P30 P31	Female, quality officer (>10) Female, resident anaesthetist (<5) Male, OR manager (>15) Male, engineer (>10) Male, engineer (>10)	Academic hospital 2 *Energy-saving measures: SB, HR, exploring RC*
Focus group 5 *on-site*	P32 P33	Female, IP specialist (>10) Male, IP specialist (<5)	Academic hospital 2
Focus group 6 *hybrid*	P34 P35 P36	Male, anaesthetist (>15) Female, gynaecologist (>15) Female, ENT surgeon (>15)	Academic hospital 1
Focus group 7 *on-site*	P37 P38 P39	Male, engineer (>15) Female, OR manager (>5) Female, IP specialist (>10)	General hospital 3 *Energy-saving measures: SB*
Focus group 8 *online*	P40 P41 P42	Male, plastic surgeon (>15) Female, GI surgeon (>15) Male, orthopaedic surgeon (>15)	Academic hospital 1

Legend: HVAC = heating, ventilation, and air conditioning; IP = infection prevention; OR = operating room; SB = setbacks outside working hours; HR = widening relative humidity setpoints; RC = reduced total volumes of air circulation; ENT = ear, nose and throat; GI = gastrointestinal

### Determinant ranking

**Fig. 1:**
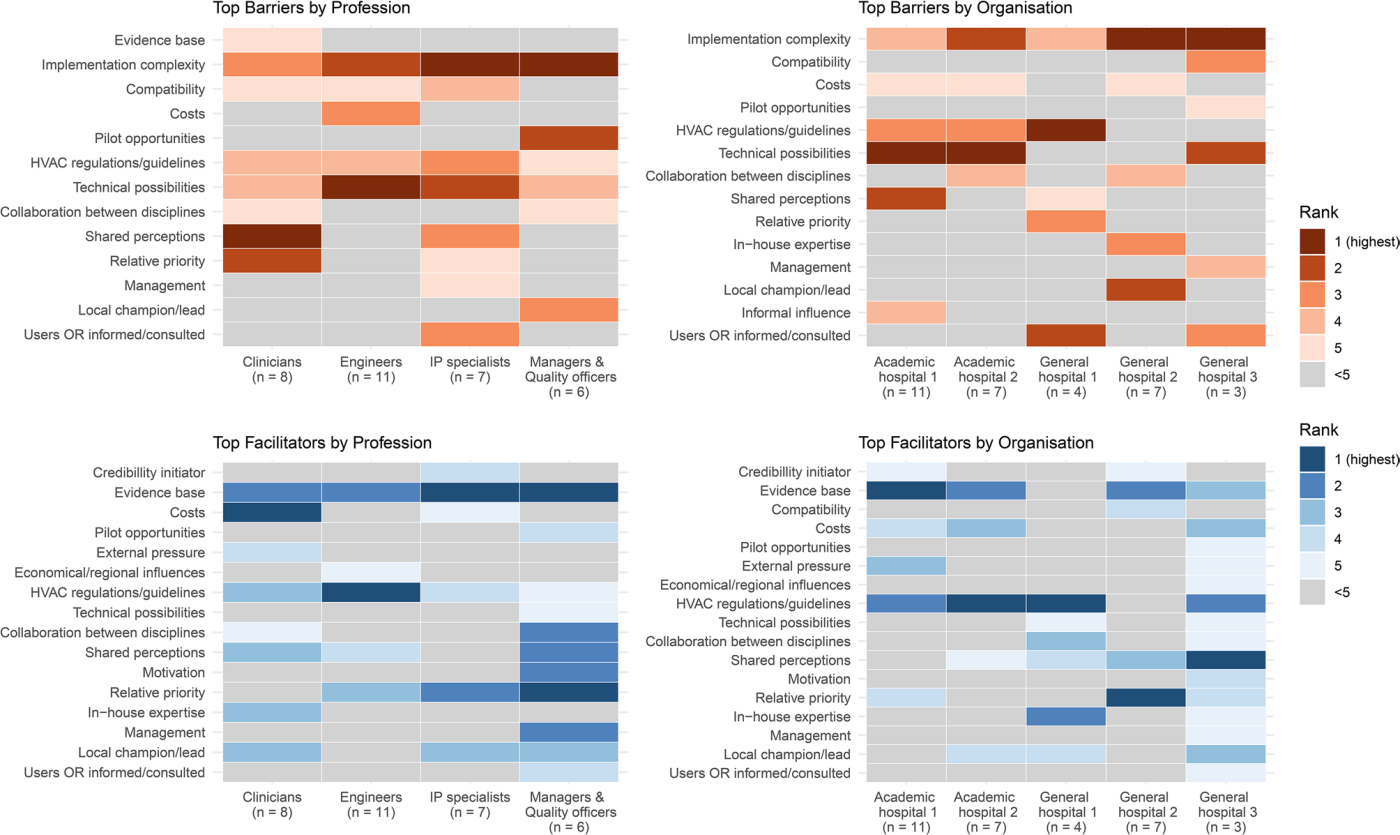
Overview of implementation determinant ranking by profession and organisation. Displayed rankings are top five ranked barriers (orange shades) and facilitators (blue shades), grouped by participants’ discipline or workplace. Only determinants included in any top five ranking are displayed. A complete, detailed overview including cumulative scores is available from Supplement C. Legend: HVAC = heating, ventilation, and air conditioning; OR = operating room; IP = infection prevention

Across settings, 32 participants ranked implementation determinants of HVAC-related energy saving measures. Overall, the top-ranked barriers were technical and organisational complexity of implementation, limitations of the hospitals’ current HVAC system, and challenging compatibility with the current way of working in the OR. The top-ranked facilitators were the evidence base for energy-saving measures and the organisation’s relative priority to save energy. Three determinants were ranked among both top barriers and facilitators: the national HVAC regulations/guidelines, the extent of shared perceptions among stakeholders regarding the importance of HVAC systems, and costs. Analysis of rankings by profession and organisation generally supported the identified top barriers and facilitators and additionally suggested a positive influence of a local champion or lead for implementation (Fig. 1). A detailed ranking overview is reported in Supplement C.

### Focus group discussions

Focus groups yielded further insights how top-ranked barriers and facilitators had influenced HVAC-related energy saving within each hospital’s context. Some hospitals were characterised by a bottom-up initiated approach, reflected in the importance of local champions to foster collaboration and in-house expertise to tailor adjustments (e.g. academic hospital 2 and general hospital 1). Other hospitals were characterised by a top-down approach which was facilitated by executive assignment or external contracting of required expertise (i.e. academic hospital 1 and general hospital 2). Participants’ disciplines were also reflected in focus group discussions, with two clinician-only sessions predominantly debating compatibility with OR workflows, patient safety, and varying perceptions whether HVAC made a relevant contribution to infection prevention at all.

**Fig. 2:**
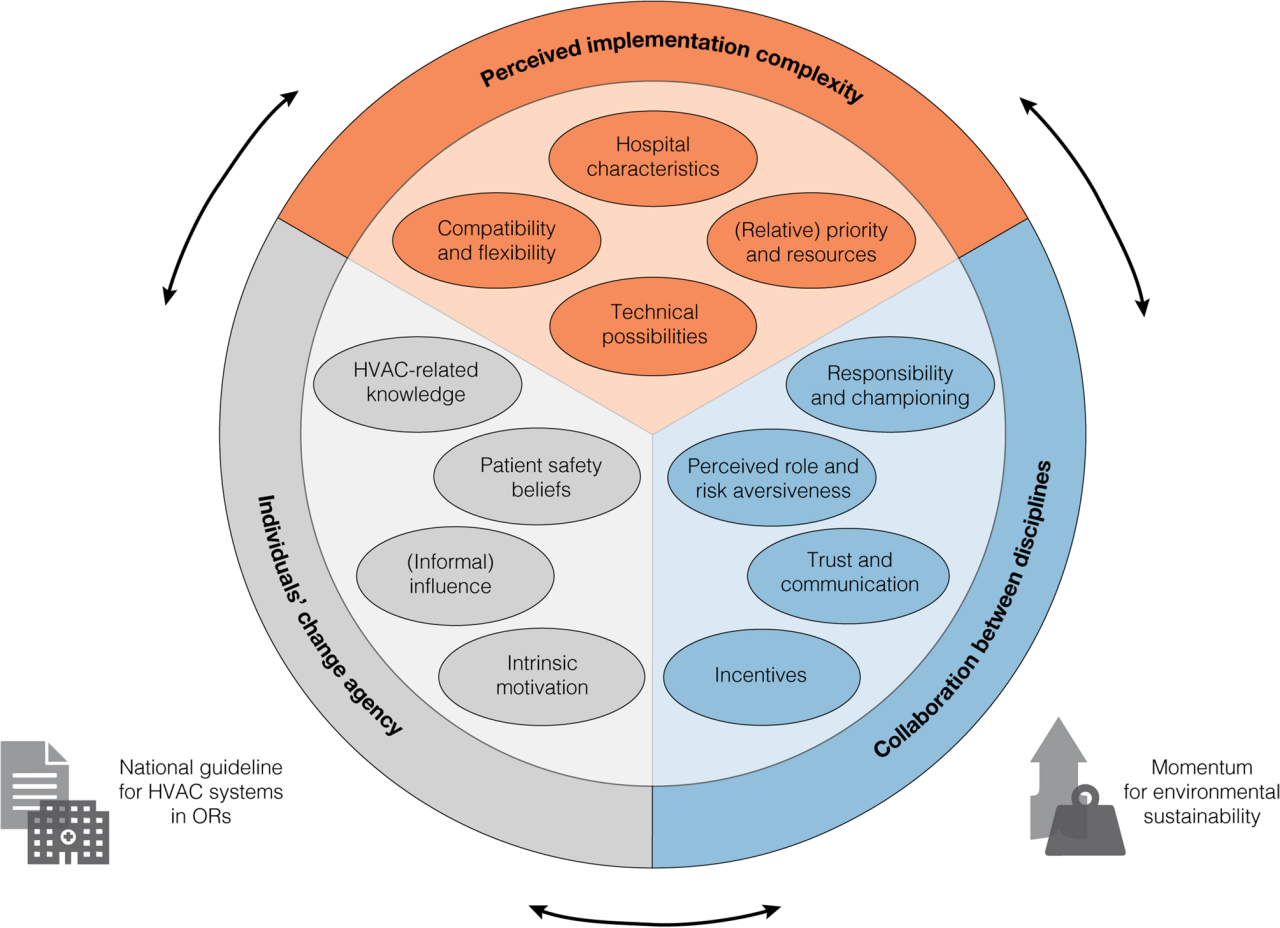
Overview of interrelated themes for top-ranked implementation determinants. Displayed findings and interrelations are based on thematic analysis of focus group findings and ranking exercise outcomes. The three overarching themes are placed in the outer circle, with sub-themes colour-coded and placed within corresponding segments. Two external factors are placed to the left and right of the thematic circle. Corresponding details and explanations are included in Table 2. Legend: HVAC = heating, ventilation, and air conditioning; OR = operating room

Overall, ranking and focus group findings could be grouped in three interrelated themes of implementation determinants: perceived implementation complexity, individuals’ change agency, and collaboration between disciplines (Fig. 2). Subtheme definitions and interrelations are detailed in Table 2.

**Table 2:** Themes for HVAC-related energy saving measure determinants and their key interrelations

**Perceived implementation complexity**		
Compatibility & flexibility	*Definition*: the degree to which the type of surgical care and preferred flexibility when scheduling surgeries are perceived to allow for implementation of HVAC-related energy saving measures.	*Interrelations*: • Larger organisational size increases the likelihood of surplus OR capacity and number of ORs exclusively available for orthopaedic major joint replacement surgery. • Shifting business focus or unclarity regarding future type of surgical care increase the need for flexibility of ORs in terms of HVAC requirements.
Technical possibilities	*Definition*: the extent to which the hospital’s current HVAC system is perceived to be technically suitable/adjustable to implement energy saving measures, such as reducing the amount of fresh outside air or maintaining consistent air conditions at reduced air circulation volumes.	*Interrelations*: • Limited knowledge regarding HVAC systems and factors influencing air cleanliness negatively influences the perceived adjustability for energy saving measures. • The national HVAC guideline specifies minimum technical requirements, yet its perception as a barrier/facilitator is influenced by the level of HVAC-related knowledge.
Hospital characteristics	*Definition*: the perceived influence of hospital characteristics on the process of implementing HVAC-related energy saving measures, including its size, decision-making style, and type of surgical care.	*Interrelations*: • Interacts with multiple complexity, collaboration, and individual themes, detailed in the corresponding sections.
(Relative) priority & resources	*Definition*: the level to which environmental sustainability or energy saving are explicit priorities compared to other (major) processes or projects and have allocated resources for HVAC-related projects or technical adjustments.	*Interrelations*: • Intrinsic motivation and growing momentum for environmental sustainability, in healthcare and in society in general, increase the likelihood for hospital leadership to prioritise HVAC-related energy saving measures.
**Collaboration between disciplines**		
Responsibility & championing	*Definition*: the perceived need for/presence of a project lead, dedicated working group, and (clinical) champion driving the implementation of HVAC-related energy saving measures.	*Interrelations*: • Resource allocation and priority for HVAC-related projects facilitates the availability of staff and (if necessary) hiring of an external project manager to lead the implementation process or make the required technical changes. • Smaller organisational size decreases the need for a dedicated HVAC working group, considering (generally) higher interconnectedness and division of responsibilities over fewer individuals within different disciplines.
Perceived role and risk aversiveness	*Definition*: the subjective role of different disciplines in advising/initiating/opposing HVAC-related energy saving measures, including a semi-normative degree of risk aversion concerning occurrence of SSIs or error-proneness of the technical system.	*Interrelations*: • Interacts with project responsibility and individual beliefs, detailed in the corresponding sections.
Trust & communication	*Definition*: the extent to which different disciplines in the same hospital regularly and successfully communicate regarding their projects/priorities and have a shared level of trust in collaboration based on previous projects.	*Interrelations*: • Presence of a person or group responsible for driving HVAC-related energy saving measures generally increases the communication and collaboration between different disciplines.
Incentives	*Definition*: the presence of (in)direct (dis)advantages for relevant disciplines to support the implementation of energy saving measures, such as financial savings, budget for workplace initiatives, or fear of medical litigation (specifically for clinicians).	*Interrelations*: • The degree to which the organisation is strictly structured in financial silos complicates the return on investment for the OR department or the use of energy expenditure savings for other workplace initiatives (i.e. shared savings). • The intrinsic motivation of relevant stakeholders influences whether additional incentives other than energy saving and environmental sustainability are necessary.
**Individual’s change agency**		
HVAC-related knowledge	*Definition*: the level of knowledge regarding HVAC systems, pertaining to technical knowledge about the functioning of the system itself as well as understanding of factors influencing air cleanliness in ORs.	*Interrelations*: • Large university hospitals are generally more likely to have in-house expertise regarding HVAC systems, both in terms of technical staff as well as among other (clinical) disciplines. • Allocation of resources to HVAC-related projects creates the possibility to hire external expertise, thereby increasing the level of HVAC-related knowledge available to the hospital.
Patient safety beliefs	*Definition*: the perception of available evidence regarding HVAC’s contribution to preventing SSIs and the consequences for patient safety when energy saving measures are implemented – including the individual’s perception of the national HVAC guideline.	*Interrelations*: • A (subjectively) risk aversive role of an individual’s discipline and limited HVAC-related knowledge generally increase the expression of patient safety concerns and need for (additional) SSI-related evidence before implementation. • Ensuring trust and communication between disciplines is important to account for patient safety beliefs, especially for clinicians who may not have an incentive to change current practice.
(Informal) influence	*Definition*: the degree to which a person has the skill, power, and/or (informal) network to engage relevant stakeholders in the hospital regarding energy saving measures and potentially practice politicking to gain implementation support.	*Interrelations*: • Ongoing organisational processes (e.g. reorganisation or new facilities) increase the likelihood of individuals using their influence to shape choices regarding HVAC systems. • Implementation success was frequently described as a combination of the right people, wherein individuals teamed based on intrinsic motivation, HVAC-related knowledge, and their (informal) power to influence decision-making.
Intrinsic motivation	*Definition*: an individual’s personal drive for environmental sustainability (in healthcare) and the time/effort they are willing to invest in driving HVAC-related energy saving measures.	*Interrelations*: • Interacts with complexity, collaboration, and individual themes, detailed in the corresponding sections.

Legend: OR = operating room; HVAC = heating, ventilation, and air conditioning; SSI = surgical site infection

#### Perceived implementation complexity

Participants differentiated between technical and organisational barriers which complicated implementation. Technical complexity was mostly addressed by engineers, discussing the adjustability of their hospital’s current HVAC system and the need for in-house expertise or investments to make necessary changes. Eventually, this required allocation of time and financial resources, dependent on managerial priority for HVAC-related projects. Implementation efforts in one hospital had long been halted due to low priority and insufficient HVAC expertise, compounded by staffing challenges in the technical department (i.e. general hospital 2). Furthermore, HVAC compatibility with clinical workflows could be complicated by unclarity regarding hospitals’ future surgical case mix or small number of ORs (e.g. general hospital 1 and 3).

P21, engineer, male, general hospital 2: The other type of complexity are the technical facilities. You’re dealing with an OR and must meet certain requirements to get it operational again. That brings another type of complexity, because you need all kinds of external companies to adjust and monitor the system.

P39, infection prevention specialist, female, general hospital 3: When you alter an OR’s HVAC settings, you create a barrier in the OR planning of not being able to perform every type of surgery in every OR. Then you must define which surgeries you perform in which OR. We currently haven’t done that.

Organisational complexity was mentioned by all participants. Generally, this included the hospital’s size and organisational structure, the need to involve different disciplines, and competing managerial priorities. The two largest hospitals had a mandated workgroup for HVAC in the OR to bring different disciplines together (i.e. academic hospitals). The smallest hospital did not consider such a structure necessary since relevant responsibilities were shared by only a few staff members (i.e. general hospital 3). Whereas all participants agreed that HVAC adjustments would yield cost reductions, several argued that a return on investment spanning multiple years was considered too long term by hospital management. Moreover, the department making the required investments may not benefit financially, limiting incentive on a departmental level.

P24, engineer, male, academic hospital 1: We purposefully created a workgroup to free up time from different disciplines. If you say: I already have too many tasks, […] it only works when it’s considered a priority. The director can say: make sure that something is taken off his plate, so he can take part.

P29, OR-manager, male, academic hospital 2: Hospitals are siloed, especially in terms of costs. You do something related to energy, but that doesn’t result in a return on investment in the OR budget - because we don’t have an energy budget. That budget is part of Facility Services and they’ll notice that suddenly their energy bill goes down. They’ll think: that’s nice!

#### Individuals’ change agency

The role of individuals was discussed in each of the focus groups. In hospitals where most energy-saving measures had been implemented (i.e. academic hospital 2 and general hospital 1), participants described this as a direct result of their shared intrinsic motivation, HVAC-related knowledge, and ability to influence decision-making. The latter required decision power, network, and/or skill. Bottom-up sustainability committees were helpful to build momentum, yet practicing politics at the right place and time had pushed actual decision-making. Engineers and infection prevention specialists in multiple hospitals regarded it less likely that anyone from their department would initiate HVAC changes, considering their advising role in the OR and frequent risk aversion for technical errors and wound infections.

P14, anaesthetist, female, general hospital 1: We’re continuously practicing politics. By now, we have managed to put sustainability higher on the agenda. But that has taken substantial effort and for several years it felt like fighting a losing battle.

P33, infection prevention specialist, male, academic hospital 2: You do need someone who takes the lead. We’d never take that role, because we’re not in charge of the OR. In that sense, we work request based. Of course, we notice the pressure related to the ‘Green Deal for Sustainable Healthcare’, […] but the most important thing is the intrinsic motivation of others to improve.

#### Collaboration between disciplines

The importance of collaboration echoed as a prerequisite throughout focus groups. Trust and communication were considered elementary to bridge varying levels of HVAC-related knowledge and beliefs regarding patient safety of HVAC adjustments. Since previous HVAC guidelines had mostly stimulated intensified HVAC operation, energy-saving measures in line with the 2022 revision had frequently been met with a certain hesitancy. Multiple participants stressed the importance to inform and involve clinicians when implementing changes to avoid conflicts – especially when introducing clean and ultra clean air OR differentiation (e.g. general hospital 1 and 3). However, clinician focus groups concluded that energy-saving measures meeting the current guideline’s standards may be implemented without extensive consultation, suggesting a decision via the OR’s medical staff board rather than extensive consultation (i.e. academic hospital 1).

P16, OR-manager, general hospital 1: In general, end user support is definitely important and you can’t just announce HVAC class changes in the newsletter. Then we’d find ourselves with the board of directors in no time to explain. Because the primary thought is: hey, that affects air quality, so it might increase our risk of infections.

P41, surgeon, academic hospital 1: I think it’s a no-brainer. Every type of surgical specialty has indicated what type of HVAC class they feel entitled to, with or without evidence. […] It’s implementation rather than change. Implement what we have agreed upon (in the guideline) and then it appears that you could do with less in several ORs.

### Implementation strategies

**Fig. 3:**
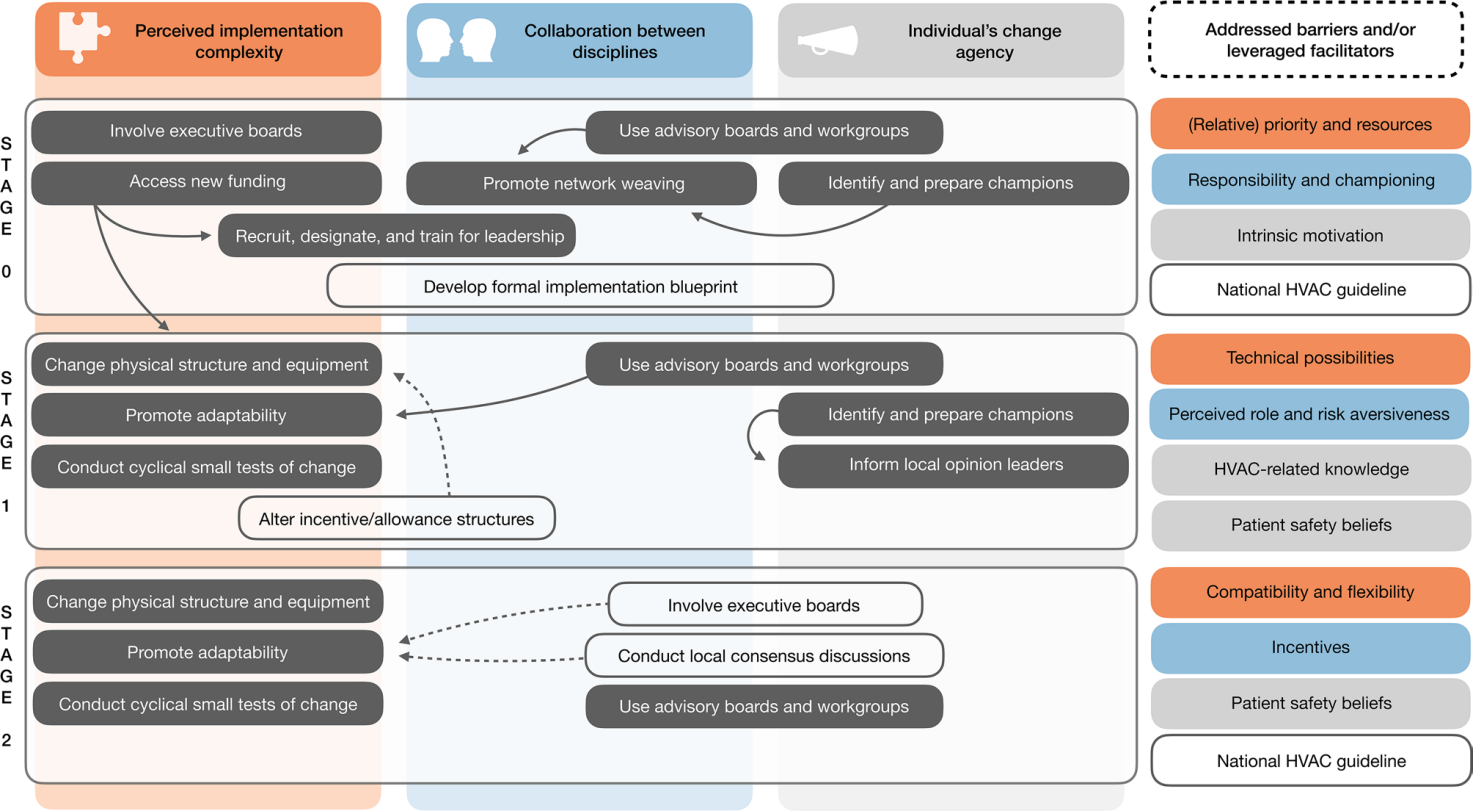
Process map of HVAC-related energy saving implementation in operating rooms. The overview includes reported (grey fill) and suggested (transparent fill) implementation strategies across HVAC energy saving implementation stages based on focus group findings and CFIR-based matching of ERIC implementation strategies. Placement of strategies and addressed barriers/facilitators indicate relevance of underlying themes and key implementation determinants. Stage 0 denotes the top-down or bottom-up implementation process initiation, stage 1 the implentation of energy-saving measures that do not affect OR workflows (e.g. setbacks and relative humidity), and stage 2 the implementation of OR differentiaton affecting workflows. Further details are available from Supplement D. Legend: HVAC = heating, ventilation, and air conditioning

Synthesis of top-ranked determinants, focus group findings, and hospitals’ progress with HVAC energy saving supported the creation of a three-stage implementation process map (Fig. 3). At ‘stage 0’, findings illustrated the prerequisite of a bottom-up or top-down initiated process to foster multidisciplinary collaboration, generate priority for energy saving, and assign responsibility for implementation. Two hospitals were currently at this stage, with one recently securing executive support and budget to hire an implementation lead (i.e. general hospital 2) and one with different managerial priorities that halted stakeholders’ time investment (i.e. general hospital 3). At ‘stage 1’, findings demonstrated the central role of a multidisciplinary workgroup to coordinate and pilot HVAC adjustments that do not directly affect OR workflows. One hospital was currently at this stage, using the workgroup’s complementary expertise to make technical adjustments and their network to obtain buy-in from colleagues and OR management (i.e. academic hospital 1). At ‘stage 2’, findings indicated the perceived importance to build consensus and leverage the most recent HVAC guideline for adjustments that affect OR workflows. Two hospitals were currently at this stage, planning to tailor OR differentiation according to clinical needs and conducting validation measurements to attest guideline-alignment (i.e. academic hospital 2 and general hospital 1). Supporting details are reported in Supplement D.

## Discussion

Implementation of HVAC-related energy-saving measures in Dutch ORs was dependent on its perceived implementation complexity, individuals’ change agency, and the degree of collaboration between disciplines. Prioritised barriers were technical and organisational implementation determinants, linked to hospitals’ size and structure, adjustability of the current HVAC system, and availability of in-house expertise. Limited HVAC-related knowledge and equivocal beliefs regarding HVAC’s contribution to infection prevention shaped perceived feasibility of energy-saving measures and compatibility with OR workflows. Prioritised facilitators were the evidence base for energy-saving measures, organisations’ relative priority to save energy, and presence of a dedicated implementation lead. Both bottom-up and top-down approaches had initiated HVAC adjustment processes in studied hospitals via close collaboration of motivated individuals and multidisciplinary workgroups.

In the wider literature, this study constitutes a novel example how environmental sustainability-focussed QI can contribute to balancing health and environmental outcomes [6, 7]. As illustrated by our HVAC-specific inquiry, targeting one of the largest sources of energy consumption and environmental impact in ORs [11, 13], an approach grounded in implementation science can provide context-rich insights in the re-evaluation of standardised OR safety measures. Excessive operation of HVAC systems should not go unchallenged when its contribution to existing infection prevention measures appears to be negligible for procedures other than arthroplasty of major joints [17, 21, 23, 24]. Assessing the reasonability of an ‘as low as reasonably practicable’ status quo would be timely and just, and need not equal throwing out the baby with the bathwater. Since such re-evaluation is likely subject to national regulatory frameworks and cultural attitudes, the setting of this Dutch study deserves specific consideration. Notwithstanding, both environmental sustainability and infection prevention (in)directly contribute to individuals’ health and healthcare quality – be it on different levels. Especially resource-intensive measures, such as HVAC systems, can therefore be valuable targets for contemporary QI initiatives.

The identified key barriers and facilitators to HVAC-related energy saving in our study are well-aligned with previous studies of complex hospital-based interventions. Organisational context, engagement of (clinical) leadership, and local champions are well-known to influence interventions’ implementation and sustainment, including infection prevention interventions in particular [37–40]. However, the marked emphasis on perceived technical and organisational complexity in our study is less common. A recent review regarding sustainment of QI programmes identified intervention complexity as a barrier in only eight out of 124 studies [41]. The reason we found perceived complexity as a barrier likely relates to the technical nature of HVAC systems, the influence of limited HVAC-related knowledge, and the need to involve multiple disciplines to make HVAC adjustments in the first place. Vice versa, evidence synthesis of QI initiatives in surgical settings reported low intervention complexity as a facilitator [42]. Our findings therefore demonstrate the importance to identify and address perceived implementation complexity, which appears particularly linked to multi-stakeholder buy-in for implementation of technical adjustments.

Pertaining to environmental sustainability in particular, our study corroborates preceding evidence for individual and organisational barriers such as staff availability, infection prevention concerns, inadequate facilities, and a lack of institutional support [43–45]. However, previous studies primarily emphasised the importance of awareness regarding healthcare’s environmental impact and sustainable practices in the OR [44, 45]. In our findings, expertise regarding HVAC and its contribution to infection prevention were more prominent than environmental awareness. Moreover, ranking outcomes demonstrated higher importance of structural determinants (i.e. technical and organisational barriers) than individuals’ awareness. While these differences may partially reflect the subject of study, they also underline the importance to avoid over-emphasis of awareness gaps to promote environmental sustainability. Recent sustainability-oriented OR studies almost exclusively focussed on clinicians to promote implementation [44–46]. Context-sensitive assessment including multiple stakeholders can provide valuable insights in more appropriate implementation strategies.

In practice, the importance of perceived complexity in this study suggests that additional strategies are necessary to promote environmental sustainability in the OR, compared to those commonly used. Previous studies primarily applied individual-focussed education and environmental restructuring to influence clinicians’ behaviour (e.g. labelling products’ environmental impact or making their placement less accessible) [47, 48]. Based on our findings, adjustment of structural processes such as HVAC require managerial priority, supported by time or budget allocation to hire external expertise and incentives for local changemakers or dedicated workgroups. We identified a key role for multidisciplinary collaboration and intrinsically motivated individuals in HVAC-related QI. Shared commitment and confidence in members’ collective ability to implement change has been theorised to determine the outcome of change initiatives [49]. While previous reviews regarding environmental sustainability in healthcare varyingly concluded a bottom-up or top-down approach to be more important [43, 44], our study underpins both points of view. When faced with too many organisational barriers (e.g. general hospital 2), individual changemakers were unable to initiate HVAC-related energy saving measures. When able to team up and strategically influence decision-making (e.g. general hospital 1), individuals managed to implement HVAC changes.

The proposed process map and corresponding examples in this study may support local changemakers or hospital organisations in selecting an implementation approach or strategies for HVAC-related energy saving. Matching of expert-recommended implementation strategies and participants’ suggestions indicates how an implementation blueprint could provide a structure and evidence-base for hospitals that still need to start HVAC energy saving. In a later implementation stage, the process map shows that guidance on how to involve medical staff boards or conduct local consensus discussions could support and attest compatibility of energy-saving measures with OR workflows. Arguably, national guideline organisations could make a valuable contribution to planning and developing such implementation strategies. Although recent evidence suggests that they infrequently engage in extensive implementation support [50], their central positioning seems particularly suitable to capture and share local knowledge or provide ongoing implementation consultation.

Strengths of this study were its combination of prioritisation and in-depth exploration of key implementation determinants in multiple hospital settings, adding valuable insights regarding interrelations and context, which are well-recognised to influence QI initiatives [51, 52]. Moreover, its focus on HVAC-related energy-saving in the OR and inclusion of non-clinician stakeholders targets an important gap in the literature.

Several limitations should be considered when interpreting the findings of this study. First, we explored experienced and anticipated implementation determinants in the participating hospitals, without longitudinal observation of HVAC-related energy saving implementation. Reported determinants were therefore not fully differentiated per energy-saving measure and could not be linked with observed measures implementation success. While we did not find evidence of such specificity in the focus group discussions and believe the inclusion of hospitals with different types of implemented energy-saving measures to offer value in its practice-aligned variability, future studies may elucidate more measure-specific linkage. Second, our five hospital settings were all situated in the Netherlands, thereby limiting international generalisation of its findings. Purposive sampling and achievement of data saturation contributed to the context-diverse nature of our findings, including different organisation structures and levels of energy saving implementation. Notwithstanding, future studies should validate our findings in different settings, such as more centralised, conservative, or litigation-dominant health systems.

## Conclusion

Our study suggests that implementation of HVAC-related energy saving measures in the OR is hampered by perceived implementation complexity, technical challenges, and individual knowledge gaps and beliefs. Dedicated multidisciplinary workgroups, managerial priority for energy-saving, and collaboration of motivated individuals with complementary expertise and influence appear to support successful local implementation. While effectiveness of implementation strategies to promote environmental sustainability in the face of risk minimisation requires further evaluation, a window to reduce HVAC’s environmental burden already appears to exist.

## Electronic supplementary material

Below is the link to the electronic supplementary material.

Supplementary material 1

## Data Availability

Materials and data directly supporting the reporting in the manuscript, including detailed ranking outcomes, implementation strategy mapping, and the coding guide, have been made available in the supplementary files and via an Open Science Framework repository https://osf.io/mexjq/overview?view_only=9a235b15c1554d379ca80780661667d8. Anonymised transcripts and original study materials (in Dutch) can be obtained from the corresponding author upon reasonable request.

## Abbreviations

QI: quality improvement; OR = operating room
HVAC: heating, ventilation, and air conditioning
CFIR: Consolidated Framework for Implementation Research
ERIC: Expert Recommendations for Implementing Change.
