# Supplementary material for: Determinants of sustainable quality improvement for operating room clean air systems: a two-phase sequential qualitative study in Dutch hospitals
Source: BMC Health Serv Res. 2026 Mar 30;26:668. doi: 10.1186/s12913-026-14358-9 (PMC13156890; doi:10.1186/s12913-026-14358-9)
Supplement: Supplementary file 1 — Supplementary material 1 [file 12913_2026_14358_MOESM1_ESM.pdf]

Supplementary material to:

# Determinants of sustainable quality improvement for operating room clean air systems: a two-phase sequential qualitative study in Dutch hospitals

Egid M. van Bree[1,2], Erin N.A. Cornelisse[2], Roberto A.A.L. Traversari[3], Evelyn A. Brakema[4], Nicole D. Bouvy[1], Rianne M.J.J. van der Kleij[4]

[1] Department of Surgery, Leiden University Medical Centre, Leiden, the Netherlands
[2] Centre for Sustainable Healthcare, Amsterdam UMC, Amsterdam, the Netherlands
[3] Organisation for Applied Scientific Research, TNO, Delft, the Netherlands
[4] Department of Public Health and Primary Care/Campus the Hague, Leiden University Medical Centre, Leiden, the Netherlands

Table of contents:

| | | |
|---|---|---|
| **Supplement A** | Study materials used for data collection | pages 2-7 |
| **Supplement B** | Shortlist of implementation determinants | page 8 |
| **Supplement C** | Determinant ranking outcomes | page 9 |
| **Supplement D** | Focus group findings and implementation strategy matching | page 11 |

## Supplement A – Study materials used for data collection

**Phase 1 – Expert interview questionnaire (translated from Dutch)**

Introduction:

In the coming period, we will organise focus groups in various hospitals to gain a better understanding of the factors that influence the implementation of energy-saving opportunities for HVAC systems in operating rooms. This is prompted by recent research conducted the HVAC expert group of the National Network for Green Operating Rooms (in Dutch: Landelijk Netwerk de Groene OK).

The focus groups will focus on the four savings opportunities identified in this recent study: 1) widening relative humidity setpoints (to 30-70%), 2) introducing setbacks to switch to a lower operating mode outside working hours, 3) reducing the amount of fresh outside air in the total air supplied to the operating room, and 4) switching the operating room class from Class 1+ to Class 1.

In preparation for the focus groups, we are conducting interviews with various experts on this topic – including yourself. The aim of these interviews is to compile as comprehensive a list as possible of influencing factors. We are not yet trying to distinguish between factors that are more or less important – we will do this in the focus groups.

After the discussions with the various experts, I will draw up a list of factors and submit it to you for verification.

Open question:

What are – based on your own experience – factors that influence the implementation of the energy saving measures for HVAC in operating rooms? [*if necessary, explicitly probe for relative humidity, setbacks, reducing fresh outside air, and class switching*]

Probing for specific CFIR domains[1]:

1. Innovation domain – factors related to the energy-saving measure itself
    a. Credibility of the initiator
    b. Sufficient evidence
    c. Adjustable to local context
    d. Possibility to pilot/test
    e. Complexity to implement (technical and organisational)
    f. Costs of the energy-saving measure

2. Outer setting – factors from outside the hospital or private clinic
    a. External events disrupting implementation
    b. External opinions/beliefs supporting the measure
    c. Political/economic conditions
    d. Collaboration with other parties
    e. Regulations and/or guidelines
    f. External financing for implementation
    g. External pressure such as societal movements, campaigns, benchmarks

[1] Note that these are constructs, freely adjusted/translated to match the subject of energy saving for HVAC in operating rooms. These are based on the revised (v2.0) Consolidated Framework for Implementation research by Damschroder et al (Implement Sci, 2022). doi: 10.1186/s13012-022-01245-0

3. Inner setting – factors from inside the hospital or private clinic
    a. Structural characteristics including physical infrastructure, IT, division of tasks
    b. Formal and informal relationships/networks/teams for collaboration
    c. Formal and informal ways to share information between stakeholders
    d. Shared values/beliefs/norms (including focus on patient wellbeing, wellbeing of employees)
    e. Degree to which the current situation cannot be maintained
    f. Degree to which the energy-saving measures are compatible with the current way of working
    g. Implementation of these energy-saving measures is considered important compared to other innovations in the operating room
    h. There are incentives or punishments which speed up or slow down the implementation of these energy-saving measures
    i. Implementation of the energy-saving measures is in line with the overarching goal of the organisation
    j. There are available resources to implement the energy-saving measures
    k. There is training or support (e.g. knowledge) available for implementation

4. Individuals – individual factors influencing implementation
    a. Role of the person(s) involved with energy-saving measures
        i. High-/mid-level leaders, opinions leaders (informal), implementation facilitators/leads, innovation deliverers/recipients
    b. Needs = implementation of the energy-saving measures fulfils a need of one or more of the persons listed above
    c. Capabilities = competency/knowledge/skills to implement the energy-saving measures
    d. Opportunities = being available, having the area of concern and the power to implement the energy-saving measures
    e. Motivation = dedication to implement the energy-saving measures (or, in general: energy saving or environmental sustainability within the organisation)

5. Implementation process – the way the energy-saving measures are implemented
    a. Are there collaborations to implement the measures
    b. Has information been collected regarding the priorities/preferences/needs of the end users of HVAC (who support the implementation process)
    c. Has the context been assessed where the measures need to be implemented
    d. Has a planning been made, have tasks been assigned
    e. Are there location-specific implementation strategies (=activities)
    f. Are the end users of HVAC involved in the implementation process
    g. Are adjustments made in small steps, or iteratively
    h. Is there reflection/evaluation of the implementation
    i. Have the energy-saving measures been adjusted to local preferences

**Phase 2 – Topic list for focus groups (translated from Dutch)**

Introduction (5-10 mins):

a. Welcome and introduction
b. Ground rules and room for questions
c. Introduction round
d. Introduction of the subject

Welcome and introduction:

Thank you for coming and participating in this discussion. I suggest we address each other informally. I will briefly introduce myself and explain the research, then we will start the discussion.

My name is [name researcher], I am a physician-researcher in the field of sustainable healthcare. This research is a collaboration between [name university] and the National Network for Green Operating Rooms. Our aim is to gain a better understanding of the factors that influence the realisation of energy-saving opportunities for HVAC systems in operating rooms. It is a follow-up to previous research in which we measured various savings options – more on that later. The reason we have invited you to this discussion is your involvement in this topic in your hospital or private clinic.

Ground rules and room for questions:

For this conversation, I would like to mention a few things:
- I am interested in your opinions and experiences – as far as I am concerned, there are no wrong answers. So feel free to mention anything that comes to mind.
- If there are things you do not understand, please let me know and I will explain them to you.
- Everything we discuss will be processed anonymously.
- The conversation will take about 1 hour.
- In order to make a record of the conversation, I would like to record it with your permission. I will delete the recording afterwards.
- You have received a consent form in advance. Could you please sign it and give it to me?

Introduction round:

Would everyone kindly introduce themselves briefly, stating their name, position, and work experience at this centre? I will not record the introductions at this time.

Introduction of the subject: [*NB: supporting slides are used, listed below on pp6-7*]

Today, we will discuss four energy-saving options for HVAC systems. Recent research by Landelijk Netwerk de Groene OK (National Network for Green Operating Rooms) has shown that these options yield the greatest savings. They are: 1) widening relative humidity setpoints (to 30-70%), 2) introducing setbacks to switch to a lower operating mode outside working hours, 3) reducing the amount of fresh outside air in the total air supplied to the operating room, 4) switching the operating room class from Class 1+ to Class 1.

As mentioned, the discussion will focus on the factors that influence the realisation of these savings opportunities. To ensure that this runs smoothly, we have done some preparatory work: together with experts, we have drawn up a list of possible facilitating and hindering factors. I have this list with me here. I will first go through it with you. After that, I will ask you to assess it individually and discuss.

Are there any questions before we get started? If not, I will start the recording.

Part 1: Discussing the shortlist and **individual** ranking of impact (± 20 mins):

[*NB: a printed version of the shortlist is given to each participant, i.e. Supplement B*]

The factors are divided into five groups. Let's go through them together.
Group 1 concerns the energy-saving opportunities themselves;
Group 2 concerns influences outside your hospital;
Group 3 concerns influences within your hospital;
Group 4 concerns person-dependent factors; and
Group 5 concerns the way in which the savings opportunities are implemented.

Before we continue: are there any factors you think are missing?

Then I would first like to ask each of you individually to indicate how important you think each of the factors is. We will do this simultaneously, in an online overview. You can enter using the QR-code on the screen.
You have a total of 100 points to distribute. You can distribute them however you like.
[*If applicable*: I will add the missing factors to the list in the meantime.]
[*NB: participants scan the QR-code and participate on the mobile device or tablet which they carry on them*]

<u>Part 2: Discussing the ranking outcomes with all participants (± 30 mins)</u>:
[*NB: the ranking outcomes are displayed live on the screen, indicating mean scores and rankings per determinant*]
Let us discuss the results together. We will do this based on the points you have given.

Possible stimulating questions:
- Would it make a big difference if this factor were remedied? Why/why not?
- Is it realistic to do something about this factor? Why/why not?
- Do you have any (recent) examples from your own experience of the relevance of these factors?
- Is this factor related to other factors? In what way?
- Can you give an example, has there been anything in the recent past..., has it always been this way for you...
- [*optionally*] We know that in other hospitals..., what is that like in your organisation?

<u>Closing (5-10 mins)</u>:
Thank you for your answers and your honesty. These were the points we wanted to discuss today. In your opinion, are there any issues that have not been discussed? Or do you have any additional comments you would like to share? […]

Within a week, I will prepare a report of the meeting with the most important outcomes and send it to you by email. Would you be willing to review it and let me know if, on further consideration, you feel that anything is missing?

Thank you very much. I will stop the recording now.

**Phase 2 – Supporting figures/slides during the focus group**

**Figure A1**. Overview of the four energy saving measures being discussed and their relative benefits (in Dutch, not translated).

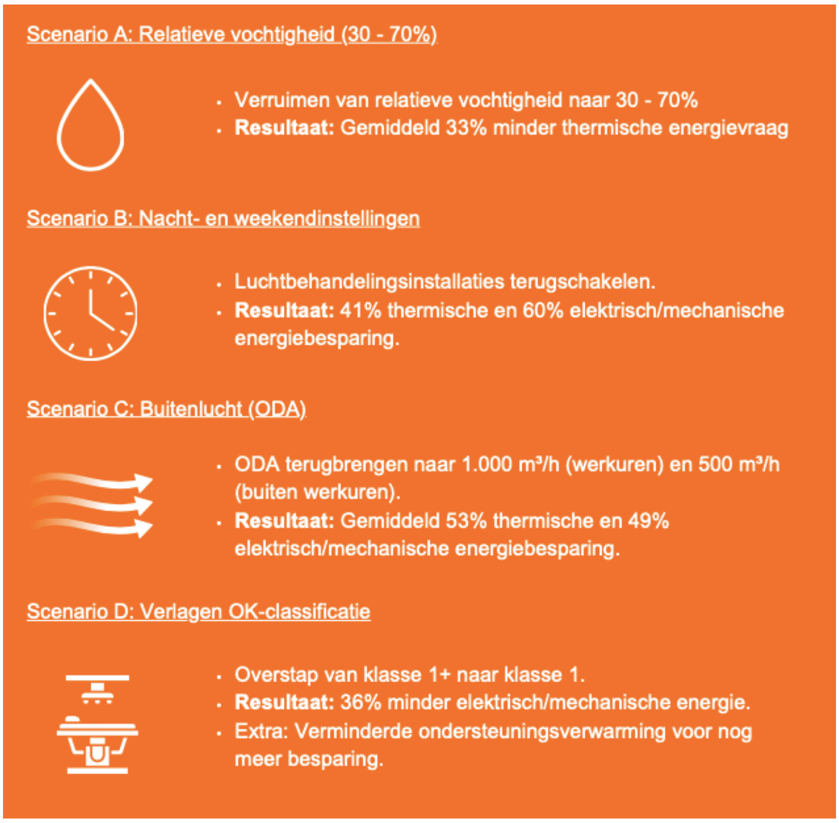


*Source: Landelijk Netwerk de Groene OK. Luchtbehandeling operatiekamers: veel potentie voor energiebesparing. Utrecht: 2025. [available from: https://degroeneok.nl/wp-content/uploads/2025/04/Eindproduct-luchtbehandeling-digitaal.pdf]*

**Figure A2**. Pie chart of the extent to which setbacks are performed in Dutch operating rooms outside of working hours, used to illustrate substantial variation in practice (in Dutch, not translated).

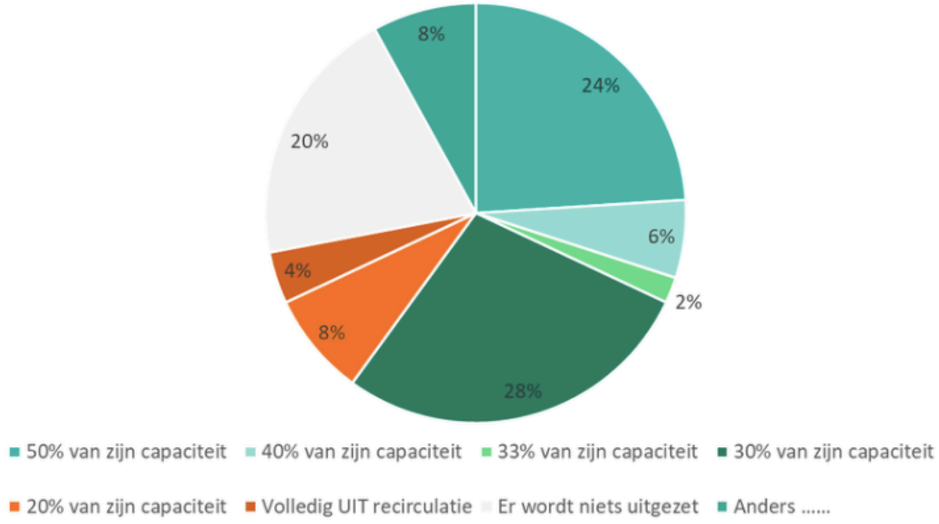


*Source: Landelijk Netwerk de Groene OK. Luchtbehandeling operatiekamers: veel potentie voor energiebesparing. Utrecht: 2025. [available from: https://degroeneok.nl/wp-content/uploads/2025/04/Eindproduct-luchtbehandeling-digitaal.pdf]*

**Figure A3**. Overview of different HVAC types and parameter complexity in management system, used to illustrate that the technical reality is more complex, but can generally be simplified into three different types of HVAC systems (in Dutch, not translated).

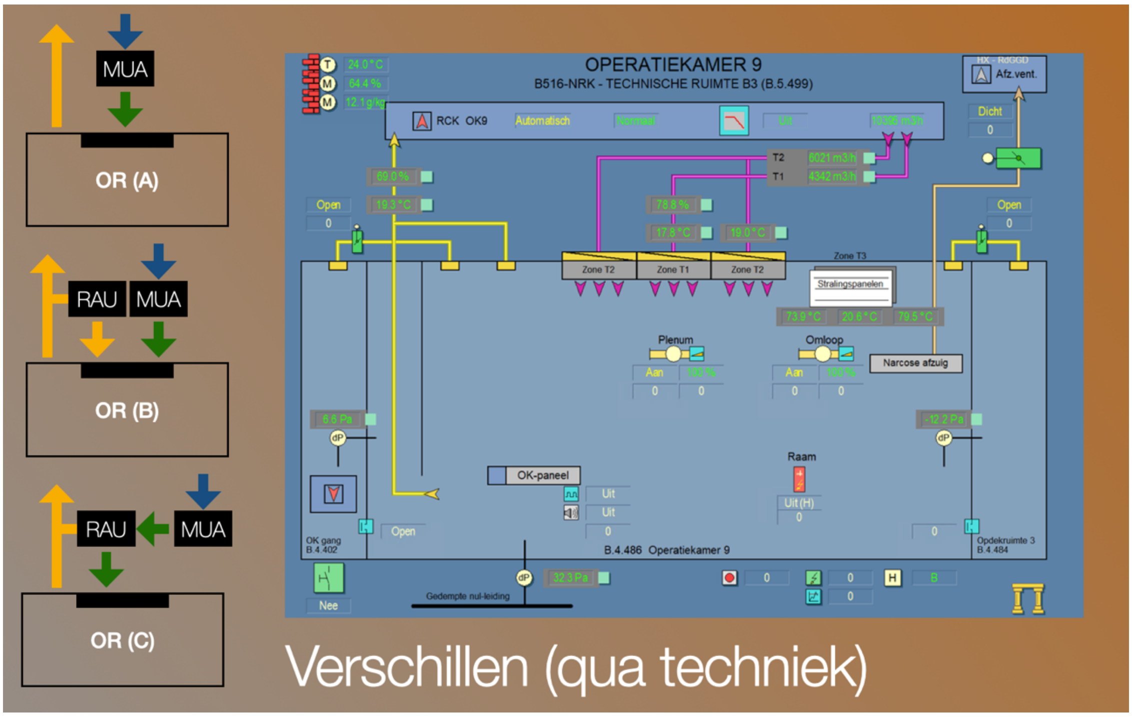


*Source: own material of the research team. Note that the simplified figures on the left-hand side of the slide represent the three general types of HVAC systems currently used in Dutch hospitals, varying in their circulation of fresh outside air that requires conditioning (i.e. 'make up air' = MUA) and recirculation of air through HEPA filters that was already in the OR (i.e. 'recirculation air units' = RAU).*

## Supplement B – Shortlist of implementation determinants

<u>Explanation for the reader:</u>
Based on discussions with 12 experts and experienced professionals (technicians, infection prevention, clinicians, management), we have compiled a list of factors that influence the implementation of energy-saving options for clean air systems in operating rooms. These include: 1) setbacks, 2) widening relative humidity setpoints, 3) reducing the amount of fresh outside air, and 4) switching operating room class.

The overview includes both barriers and facilitators. It should be noted that the absence of a barrier does not necessarily equate to a facilitator. These are factors that either actively hinder implementation or help to facilitate it. The factors are divided into groups according to where they apply.
*Please let us know if you think anything is missing.*

**Group 1 – The energy saving potential**
1. Credibility/reliability of the initiator
2. Evidence base for the energy saving measure(s)
3. Technical and organisational complexity of implementation
4. Extent to which the energy-saving measure is compatible with current working practices
5. Costs and financial feasibility (e.g. initial investment versus savings)
6. Local applicability and testing/pilot opportunities

**Group 2 – Influences from outside the hospital**
7. External pressure to become more sustainable/save energy (e.g. politics, hospital image)
8. Economic or regional influences to save energy (e.g. electricity grid congestion)
9. Degree of collaboration with other hospitals or networks (e.g. Network for Green Operating Rooms)
10. National HVAC regulations and/or guidelines (e.g. Health and Safety Guideline 2010, FMS Guideline 2022)[2]

**Group 3 – Influences within the hospital**
11. Technical facilities (possibilities/limitations of the current HVAC system)
12. Distribution of tasks and responsibilities within the organisation
13. Degree of collaboration and communication between different disciplines
14. Extent to which those involved share perceptions regarding (the importance of) HVAC systems (e.g. SSIs, comfort in the operating room, sustainability)
15. Motivations/rewards for the department to realise energy-saving measures
16. (Relative) priority of the organisation to save energy (cost savings, sustainability)
17. Degree of in-house expertise regarding HVAC systems

**Group 4 – Role of individuals**
18. Extent to which management or department heads have the knowledge/motivation/opportunity to implement energy-saving measures
19. Extent to which there is a local champion/implementation lead who has the knowledge/motivation/opportunity to implement energy-saving measures
20. Extent to which influential individuals have an informal influence on implementation

**Group 5 – Actual implementation**
21. Action plan in which tasks and responsibilities are divided
22. Extent to which end users/employees in the OR have been consulted/informed

*Legend: HVAC = heating, ventilation, and air conditioning; SSI = surgical site infection; OR = operating room*

[2] Note that these guidelines refer to Dutch guidelines regarding health and safety at the workplace (in Dutch: "arborichtlijn") and the guideline of the Dutch Federation of Medical Specialists (i.e. FMS), which details requirements for HVAC systems in operating rooms and specifies which surgeries should take place in which 'class' of operating room.

## Supplement C – Determinant ranking outcomes

**Table C1**. Ranking of shortlisted implementation determinants as barriers across focus groups

| Determinant | Computed total participant scores (n=32) | Occurrence in top 5 per hospital (n=5) | Occurrence in top 5 per profession (n=4) |
|---|---|---|---|
| 1. Credibility of initiator | 97 | - | - |
| 2. Evidence base for the energy saving measure(s) | 139 | - | C |
| 3. Technical and organisational complexity of implementation | 335 | A1,A2,G1,G2,G3 | C,E,IP,M |
| 4. Compatibility with current way of working | 196 | G3 | C,E,IP |
| 5. Costs and financial feasibility | 189 | A1,A2,G2 | E |
| 6. Applicability and pilot opportunities | 143 | G3 | M |
| 7. External pressure to save energy | 26 | - | - |
| 8. Economical/regional influences to save energy | 53 | - | - |
| 9. Collaboration with other hospitals/networks | 61 | - | - |
| 10. National HVAC regulations and/or guidelines | 243 | A1,A2,G1 | C,E,IP,M |
| 11. Technical possibilities of current HVAC system | 297 | A1,A2,G3 | C,E,IP,M |
| 12. Distribution of tasks and responsibilities | 115 | - | - |
| 13. Collaboration between disciplines | 141 | A2,G2 | C,M |
| 14. Shared perceptions regarding (the importance of) HVAC | 189 | A1,G1 | C,IP |
| 15. Motivation for energy-saving measures | 63 | - | - |
| 16. (Relative) priority of energy saving | 156 | G1 | C,IP |
| 17. In-house expertise | 94 | G2 | - |
| 18. Management knowledge/motivation/ability to implement | 117 | G3 | IP |
| 19. Presence of local champion/lead | 104 | G2 | M |
| 20. Informal influence on implementation | 107 | G1 | - |
| 21. Presence of action plan | 78 | - | - |
| 22. Users in the OR consulted/informed | 127 | G1,G3 | IP |

*Data are computed total participant scores (i.e. total number of points awarded to a determinant by all participants) and the occurrence of determinants as top 5 ranked barrier across focus groups (i.e. based on the total number of points per hospital or per profession). Hospitals are academic hospitals (A1-2) and general hospitals (G1-3). Professions are clinicians (C), engineers (E), infection prevention specialists (IP), and operating room managers or quality officers (M). Coloured cells indicate rankings from 1 (=highest, dark shade) to 5 (light shade). Legend: HVAC = heating, ventilation, and air conditioning system; OR = operating room.*

*Note: underlying participant data are available from the Open Science Framework (link). Participants mostly awarded 0 points (389x), 10 points (125x), 5 points (53x), or 20 points (36x). The maximum number of points awarded was 30 – which occurred no more than twice per barrier and 11 times in total.*

**Table C2**. Ranking of shortlisted implementation determinants as facilitators across focus groups

| Determinant | Computed total participant scores (n=32) | Frequency in top 5 per hospital (n=5) | Frequency in top 5 per profession (n=4) |
|---|---|---|---|
| 1. Credibility of initiator | 117 | A1,G2 | IP |
| 2. Evidence base for the energy saving measure(s) | 301 | A1,A2,G2,G3 | C,E,IP,M |
| 3. Technical and organisational complexity of implementation | 21 | - | - |
| 4. Compatibility with current way of working | 90 | G2 | - |
| 5. Costs and financial feasibility | 172 | A1,A2,G3 | C,IP |
| 6. Applicability and pilot opportunities | 108 | G3 | M |
| 7. External pressure to save energy | 171 | A1,G3 | C |
| 8. Economical/regional influences to save energy | 102 | G3 | E |
| 9. Collaboration with other hospitals/networks | 28 | - | - |
| 10. National HVAC regulations and/or guidelines | 279 | A1,A2,G1,G3 | C,E,IP,M |
| 11. Technical possibilities of current HVAC system | 113 | G1,G3 | M |
| 12. Distribution of tasks and responsibilities | 54 | - | - |
| 13. Collaboration between disciplines | 149 | G1,G3 | C,M |
| 14. Shared perceptions regarding (the importance of) HVAC | 189 | A2,G1,G2,G3 | C,E,M |
| 15. Motivation for energy-saving measures | 136 | G3 | M |
| 16. (Relative) priority of energy saving | 236 | A1,G2,G3 | E,IP,M |
| 17. In-house expertise | 148 | G1,G3 | C |
| 18. Management knowledge/motivation/ability to implement | 109 | G3 | M |
| 19. Presence of local champion/lead | 150 | A2,G1,G3 | C,IP,M |
| 20. Informal influence on implementation | 50 | - | - |
| 21. Presence of action plan | 62 | - | - |
| 22. Users in the OR consulted/informed | 103 | G3 | M |

*Data are computed total participant scores (i.e. total number of points awarded to a determinant by all participants) and the occurrence of determinants as top 5 ranked facilitator across focus groups (i.e. based on the total number of points per hospital or per profession). Hospitals are academic hospitals (A1-2) and general hospitals (G1-3). Professions are clinicians (C), engineers (E), infection prevention specialists (IP), and operating room managers or quality officers (M). Coloured cells indicate rankings from 1 (=highest, dark shade) to 5 (light shade). Legend: HVAC = heating, ventilation, and air conditioning system; OR = operating room.*

*Note: underlying participant data are available from the Open Science Framework (link). Participants mostly awarded 0 points (365x), 10 points (152x), 5 points (40x), or 20 points (29x). The maximum number of points awarded was 40 – which occurred only once for determinant 10.*

## Supplement D – Focus group findings and implementation strategy matching

**Figure D1**. (Re)mapping of barriers/facilitators across focus groups to CFIR domains

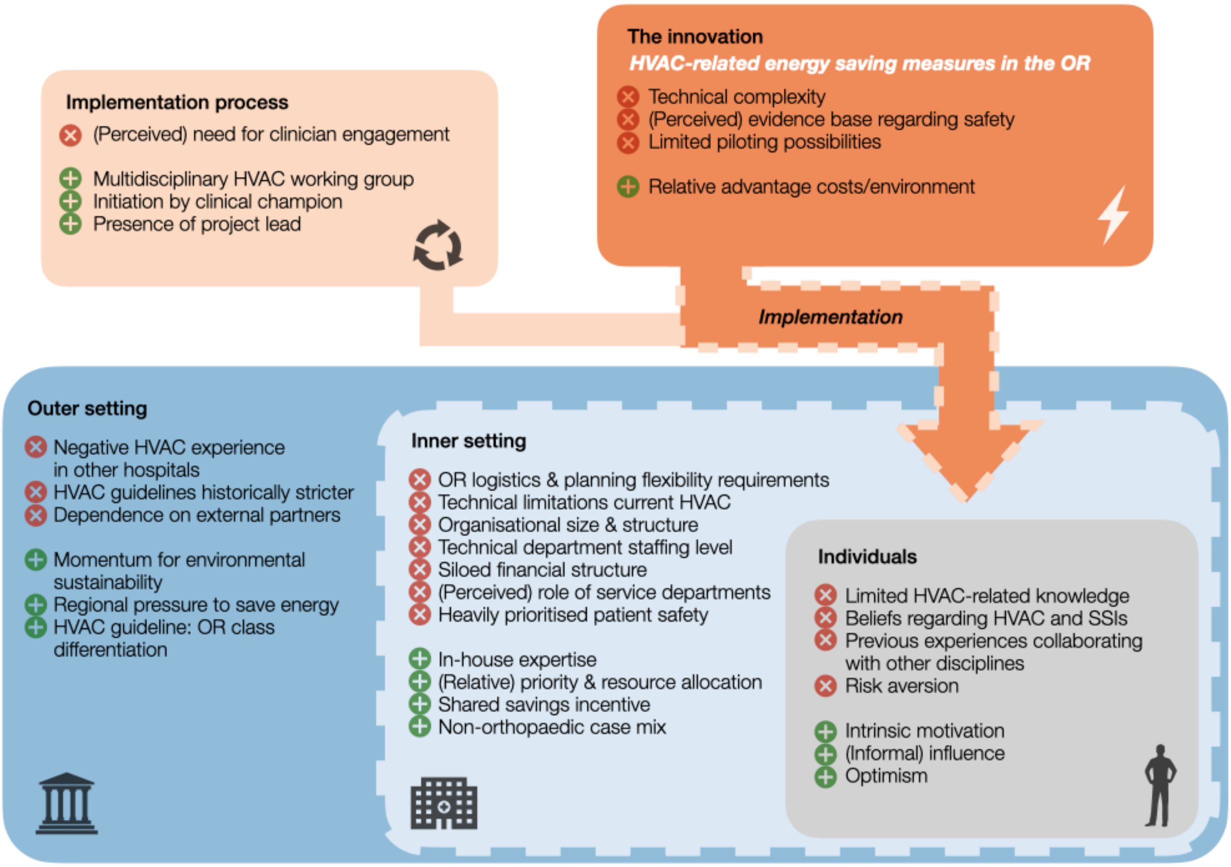


Legend: HVAC = heating, ventilation, and air conditioning system; OR = operating room; SSI = surgical site infection
⊗ denotes barrier to implementation; ⊕ denotes facilitator to implementation

**Table D1**. (Re)matching of top-ranked barriers to CFIR constructs and ERIC implementation strategies

| **Top-ranked barrier (highest to lowest)** | **Underlying CFIR 2.0 construct *** | **Level 1 ERIC strategy §** | **Level 2 ERIC strategy §** |
|---|---|---|---|
| Technical and organisational complexity of implementation | Innovation complexity (I.A) | none | **- Develop an implementation blueprint**<br>**- Promote adaptability**<br>**- Conduct cyclical small tests of change**<br>- Create a learning collaborative<br>**- Identify and prepare champions**<br>- Assess for readiness and identify barriers/facilitators<br>- Tailor strategies<br>- Provide ongoing consultation<br>- Implementation facilitation |
| Technical possibilities of current HVAC system | Structural characteristics (III.A) | none | - Assess for readiness and identify barriers/facilitators<br>**- Change physical structure and equipment**<br>**- Identify and prepare champions**<br>- Build a coalition<br>- Capture and share local knowledge<br>**- Conduct cyclical small tests of change**<br>**- Promote adaptability**<br>**- Promote network weaving** |
| National HVAC regulations and/or guidelines | Policies and laws (II.E) | n/a ‡ | n/a ‡ |
| Compatibility with current way of working | Compatibility (III.F) | none | **- Promote adaptability**<br>**- Conduct local consensus discussions**<br>**- Conduct cyclical small tests of change**<br>- Tailor strategies<br>- Assess for readiness and identify barriers/facilitators<br>- Implementation facilitation<br>**- Identify and prepare champions**<br>- Build a coalition<br>- Conduct a local needs assessment |
| Shared perceptions regarding (the importance of) HVAC | Culture (III.D) | **Identify and prepare champions** | - Assess for readiness and identify barriers/facilitators<br>**- Recruit, designate, and train for leadership**<br>- Create a learning collaborative<br>- Facilitation<br>- Tailor strategies<br>- Capture and share local knowledge<br>- Conduct educational meetings<br>**- Conduct local consensus discussions**<br>- Conduct local needs assessment<br>**- Inform local opinion leaders**<br>**- Use advisory boards and workgroups** |
| Costs and financial feasibility | Innovation cost (I.H) | **Access new funding** | - Alter incentive/allowance structures<br>- Develop resource sharing agreements<br>**- Involve executive boards** |

*Overview of top-ranked barriers and suggested implementation strategies to address them. Based on focus group discussions, strategies are indicated that were applied (bold, blue) or suggested (bold, dark orange). Legend: HVAC = heating, ventilation, and air conditioning system; CFIR = consolidated framework for implementation research; ERIC = expert recommendations for implementing change.*

** Based on Damschroder et al. (Implement Sci. 2022, doi: 10.1186/s13012-022-01245-0).*
*§ Based on the updated CFIR 2.0 – ERIC matching tool, originally described by Waltz et al. (Implement Sci. 2019, doi: 10.1186/s13012-019-0892-4). Level 2 strategies are listed in descending order of importance. Strategies that do not apply to HVAC energy saving were omitted (e.g. "model and simulate change" or "identify early adopters").*
*‡ This construct was newly added to the CFIR 2.0 and therefore not included in the original CFIR – ERIC matching tool publication.*

**Table D2**. Occurrence of ERIC implementation strategies across focus group discussions

| ERIC strategy * | Hospital | Elaboration § |
|---|---|---|
| Identify and prepare champions | A2,G1 | Relates to: *Responsibility and championing*<br>Implementation had taken flight due to advocacy of one or more intrinsically motivated stakeholders (e.g. clinician, OR manager) |
| | G3 | Relates to: *Responsibility and championing*<br>Absence of a local champion with the skillset and overview to drive implementation |
| Access new funding + Involve executive boards | A1,A2,G2 | Relates to: *(Relative) priority and resources*<br>Dedicated time or budgeting for the technical department to support implementation due to increased executive priority |
| Develop an implementation blueprint | G1 | Relates to: *National HVAC guideline*<br>Participants suggested that a formal clarification elaborating on guideline-conformity of energy-saving measures would be helpful |
| | G2 | Relates to: *Responsibility and championing; HVAC-related knowledge*<br>Participants stated the importance to develop a (local) blueprint for implementation, especially in case of staff turnover |
| Conduct cyclical small tests of change | A2 | Relates to: *Technical possibilities; Patient safety beliefs*<br>Pilot testing had confirmed to what extent HVAC setbacks outside working hours could be performed |
| | G3 | Relates to: *Technical possibilities; Patient safety beliefs*<br>Participants suggested that pilot testing would be required yet was complicated by high OR occupancy and incurred costs of downtime |
| Change physical structure and equipment + Promote adaptability | A2 | Relates to: *Technical possibilities; Compatibility and flexibility*<br>ORs were successfully retrofitted with motion sensors to support HVAC setbacks outside working hours |
| | A1,A2,G1,G3 | Relates to: *Technical possibilities; Compatibility and flexibility*<br>Participants in multiple hospitals suggested creating dedicated groups of ORs which could have ultra clean air for orthopaedic surgery; two hospitals had started to explore implementation |
| Conduct local consensus discussions + Involve executive boards | G1,G3 | Relates to: *Compatibility and flexibility; Patient safety beliefs*<br>Participants suggested consulting surgical staff regarding acceptability of OR class differentiation |
| | A1 | Relates to: *Compatibility and flexibility; Patient safety beliefs*<br>Participants suggested consulting surgical staff regarding acceptability; clinicians stated that consultation via representatives or a medical staff board would suffice |
| Recruit, designate, and train for leadership | G2 | Relates to: *Responsibility and championing*<br>The hospital had recently hired an external project lead (with relevant experience) to coordinate implementation |
| Inform local opinion leaders | A1,G1 | Relates to: *Informal influence*<br>Participants strategically identified stakeholders to inform or convince to facilitate implementation and/or pilot tests |
| Use advisory boards and workgroups + Promote network weaving + Promote adaptability | A1,A2 | Relates to: *Responsibility and championing; Trust and communication; HVAC-related knowledge*<br>Presence of a formal multidisciplinary HVAC working group to collaborate and coordinate implementation |
| | G1 | Relates to: *Responsibility and championing; HVAC-related knowledge; Intrinsic motivation*<br>Participants had created an informal multidisciplinary working group to collaborate on implementation |
| Alter incentive/allowance structures | A1,A2 | Relates to: *Incentives; (Relative) priority and resources*<br>Participants suggested a 'shared savings' approach to re-allocate energy bill savings to the OR budget |

*Overview of ERIC strategies and their occurrence as applied strategies (shaded blue) or suggested strategies (shaded orange) in hospital focus group discussions. Hospitals are academic hospitals (A1-2) and general hospitals (G1-3). Legend: ERIC = expert recommendations for implementing change; HVAC = heating, ventilation, and air conditioning system; OR = operating room.*

** Selection based on matching of top-ranked barriers and ERIC strategies (Supplementary Table D1)*

*§ Indicates how a strategy relates to the interrelated key themes included in main text Figure 2*
